# A commentary on ‘Neurosurgical application of pineal region tumor resection with 3D 4K exoscopy via infratentorial approach: a retrospective cohort study’

**DOI:** 10.1097/JS9.0000000000001038

**Published:** 2024-01-11

**Authors:** Jiahua Zhou, Di Yang, Dayun Feng, Huaizhou Qin, Chao Wang

**Affiliations:** a Department of Neurosurgery; b Department of Radiology, Tangdu Hospital, Air Force Medical University, Xi’an, Shanxi, People’s Republic of China

*Dear Editor,*

Given that pineal area tumors are situated in the geometric center of the brain and are encircled by vital neurovascular structures, aggressive surgical resection – the cornerstone of care in nearly all cases – remains technically difficult^1^. Hua *et al*.^2^. performed a retrospective cohort study on patients with pineal area lesions who had three-dimensional exoscopic tumor resection. It appears that the exoscope’s specific superiority for identified pineal area malignancies should be underlined.

The 25 patients in this retrospective analysis were acquired between April 2018 and November 2021, comprising 16 males and 9 females. The malignancies in the pineal area were excised endoscopically using the supra cerebellar infratentorial technique in all cases. In 17 patients, external ventricular drainage (EVD) with Ommaya reservoir implantation was performed before tumor excision. The results of the study showed that all patients’ postoperative hydrocephalus was greatly improved, and four patients who had relapsed hydrocephalus were cured during the long-term follow-up. A mean follow-up of 24.8±14.3 months suggested a satisfactory outcome, and postoperative adjuvant therapy was advised for appropriate patients.

However, there were still a lot of restrictions. First, some residual confounding factors may not have been considered. The male–female ratio, age distribution, and different tumor types may reduce the correct assessment of the efficiency of the operation. As is widely acknowledged, tumors of the pineal region (PRT) are extremely uncommon, making up less than 1% of all intracranial tumor lesions. PRT tumors include primary pineal parenchymal tumors, s (GCTs), and tumors that originate from nearby structures, such as meningiomas, gliomas, and choroid plexus tumors^3,4^. If more cases can be identified, analysis of indications and risk factors in diverse patient subgroups is suitable. Second, since this is a retrospective study, there may be potential bias. Finally, the bias exists since there is no control group. Control group options include conventional microscopy occipital transtentorial approach (OTA) and endoscopic OTA, which are the most widely used in the treatment of pineal tumors. Additionally, the removal of pineal area tumors may be accomplished with the endoscopic OTA, which offers the benefits of endoscopic surgery, such as less invasiveness, a brilliant panoramic magnified image, and the absence of blind spots^5^. Confirming this result will require a multicenter and more thorough investigation. In addition, additional prospective studies, clinical trials, and indication reviews are necessary to confirm the outcomes of this technique with a broad patient population to clarify its safety and efficacy.

This study describes and evaluates the benefits and limits of using exoscopes in the resection of indicated PRTs. This issue has enormous ramifications for clinical practice. This retrospective cohort study provides preliminary evidence that the exoscope is a useful tool for PRT resection and hydrocephalus relief, laying the groundwork for larger, multicenter trials. This innovative strategy offers insightful direction and motivation for the ongoing advancement of this field of study. To summarize, this study provides preliminary evidence that the exoscope is a useful tool for PRT resection and hydrocephalus relief, particularly with posterior third ventricle invasion, because total resection was possible without obvious complications, laying the groundwork for larger studies. To emphasize the unique advantage of exoscope for indicated pineal area malignancies, more studies and research should be done.

## Ethical approval

This manuscript is a comment. Don’t need ethical approval.

## Consent

This manuscript is a comment. Don’t need patient consent.

## Sources of funding

Not applicable.

## Author contribution

J.Z. and D.Y.: study concept or design, data collection, data analysis or interpretation, and writing the paper; D.F. and H.Q.: data collection; C.W.: study concept or design, writing, and revising the paper.

## Conflicts of interest disclosure

This manuscript is a comment without conflicts of interest.

## Research registration unique identifying number (UIN)

This manuscript is a comment. Don’t need UIN.

## Guarantor

Chao Wang.

## Data availability statement

This manuscript is a comment. Don’t need a Data availability statement. However, all the data from the current study are publicly available.

## Provenance and peer review

This manuscript is a comment without being invited.
